# Enteropathogenic *Escherichia coli* Infection Induces Diarrhea, Intestinal Damage, Metabolic Alterations, and Increased Intestinal Permeability in a Murine Model

**DOI:** 10.3389/fcimb.2020.595266

**Published:** 2020-12-17

**Authors:** Solanka E. Ledwaba, Deiziane V. S. Costa, David T. Bolick, Natasa Giallourou, Pedro H. Q. S. Medeiros, Jonathan R. Swann, Afsatou N. Traore, Natasha Potgieter, James P. Nataro, Richard L. Guerrant

**Affiliations:** ^1^Department of Microbiology, University of Venda, Thohoyandou, South Africa; ^2^Department of Physiology and Pharmacology, Federal University of Ceará, Fortaleza, Brazil; ^3^Center for Global Health, Division of Infectious Disease and International Health, University of Virginia School of Medicine, Charlottesville, VA, United States; ^4^Faculty of Medicine, Department of Metabolism, Digestion and Reproduction, Imperial College, London, England; ^5^Institute of Biomedicine, Federal University of Ceará, Fortaleza, Brazil; ^6^Department of Pediatrics, University of Virginia School of Medicine, Charlottesville, VA, United States

**Keywords:** enteropathogenic *Escherichia coli*, murine model, diarrhea, enteropathy, antibiotics, inflammation

## Abstract

Enteropathogenic *E. coli* (EPEC) are recognized as one of the leading bacterial causes of infantile diarrhea worldwide. Weaned C57BL/6 mice pretreated with antibiotics were challenged orally with wild-type EPEC or *escN *mutant (lacking type 3 secretion system) to determine colonization, inflammatory responses and clinical outcomes during infection. Antibiotic disruption of intestinal microbiota enabled efficient colonization by wild-type EPEC resulting in growth impairment and diarrhea. Increase in inflammatory biomarkers, chemokines, cellular recruitment and pro-inflammatory cytokines were observed in intestinal tissues. Metabolomic changes were also observed in EPEC infected mice with changes in tricarboxylic acid (TCA) cycle intermediates, increased creatine excretion and shifts in gut microbial metabolite levels. In addition, by 7 days after infection, although weights were recovering, EPEC-infected mice had increased intestinal permeability and decreased colonic claudin-1 levels. The *escN *mutant colonized the mice with no weight loss or increased inflammatory biomarkers, showing the importance of the T3SS in EPEC virulence in this model. In conclusion, a murine infection model treated with antibiotics has been developed to mimic clinical outcomes seen in children with EPEC infection and to examine potential roles of selected virulence traits. This model can help in further understanding mechanisms involved in the pathogenesis of EPEC infections and potential outcomes and thus assist in the development of potential preventive or therapeutic interventions.

## Introduction

Gastroenteritis remains a major cause of morbidity and mortality in young children especially in developing countries (Liu et al., 2015). Enteropathogenic *E. coli* (EPEC) has been recognized by the Global Enteric Multicenter Study (GEMS) and Malnutrition and Enteric Disease (MAL-ED) studies as one of the major causes of moderate to severe diarrhea in children (Kotloff et al., 2013; Platts-Mills et al., 2015). Infection results in acute watery diarrhea accompanied by fever, vomiting and dehydration (Kaper et al., 2004; Guerrant et al., 2011).

EPEC contains the locus of enterocyte effacement regulator (*Ler*) gene, a major transcriptional activator of LEE pathogenicity island, comprising around 41 open reading frames (Frankel et al., 1998; Friedberg et al., 1999). EPEC virulence is mediated by the Type 3 secretion system (T3SS), characterized by attaching and effacing (A/E) lesions (Frankel et al., 1998). The T3SS consists of EPEC secreted components (Esc) and EPEC secretion proteins (Esp), encoded in the LEE pathogenicity island. In addition, *EscN* is the main driving force assisting in the ATPase response to enable activation of the T3SS, for efficient transportation of effector proteins into the enterocytes of the host (Andrade et al., 2007). During infection, EPEC attaches to epithelial cells *via* bundle forming pili (*bfp*) (Giron et al., 1991) followed by intimate adherence with the aid of the translocated intimin receptor (*tir*) and intimin (*eae*), which results in actin accumulation and formation of pedestal structures (Knutton et al., 1989; Guerrant et al., 2011). EPEC is characterized by the presence (typical EPEC) or absence (atypical EPEC) of *bfp*. Typical EPEC are characterized by Localized Adherence (LA) *in vitro* (Kaper et al., 2004) and have been reported to cause severe diarrhea in children under 12 months of age and in certain cases results in death (Kotloff et al., 2013; Platts-Mills et al., 2015). Atypical EPEC is characterized by LA-like (Scaletsky et al., 2010), aggregative adherence or diffuse adherence *in vitro* (Pelayo et al., 1999; Mora et al., 2009) and are increasingly being detected in children worldwide (Abe et al., 2009; Hu and Torres, 2015).

Pathogens such as EPEC compete with the resident microbiota for nutrients in order to colonize the intestinal environment. According to Freter’s nutrient niche, in order for microbes to be successful, they must have the capacity to grow fast in the intestine compared to its competitors (Freter et al., 1983). These pathogens require the same carbon pathways which commensal *E. coli* uses, such as mannose and galactose *in vivo* (Fabich et al., 2008).

EPEC have been studied extensively *in vitro*, which enables studies of localization traits, A/E lesions and expression of the T3SS effector proteins (Giron et al., 1991; Knutton et al., 1999; Leverton and Kaper, 2005). *In vivo* studies have shown that a complete intestinal environment helps further determine the specific roles of EPEC traits involved in infections in animals and humans (Law et al., 2013). Animal models such as *Caenorhabditis elegans*, rabbits, pigs, and cattle have been used to study EPEC infections (Moon et al., 1983; Larsen et al., 1995; Dean-Nystrom et al., 2002; Misyurina et al., 2010). Infections induced by EPEC in C57BL/6 mice have also been reported (Savkovic et al., 2005; Royan et al., 2010; Zhang et al., 2010; Rhee et al., 2011; Manthey et al., 2014; Dupont et al., 2016), showing colonization of EPEC in the intestinal epithelial microvilli (Savkovic et al., 2005), changes in tight junction morphology and epithelial barrier function accompanied by inflammatory responses (Zhang et al., 2010; Zhang et al., 2012).

Most of the previous EPEC infection murine models have used streptomycin in order to disrupt the intestinal microbiota and promote colonization in mice (Mundy et al., 2006; Zhang et al., 2010; Rhee et al., 2011; Zhang et al., 2012; Dupont et al., 2016). Recently, a study demonstrated that mice are susceptible to colonization with EPEC in an age and microbiota disruption-dependent manner, with infant (preweaning) mice being more susceptible (Dupont et al., 2016). These animal models have provided insights into the understanding of potential pathogenetic mechanisms of EPEC infection in humans. However, these models have not been able to replicate clinical outcomes observed in humans (growth decrement and diarrhea).

A murine EPEC infection model able to induce changes in body weight and diarrhea, which are important outcomes in children infected by EPEC, had been needed and was the focus of our current study (Law et al., 2013). We have previously shown that disruption of intestinal microbiota using a broad-spectrum antibiotic cocktail enabled colonization of bacterial pathogens such as Enterotoxigenic *E. coli* (Bolick et al., 2018), *Campylobacter jejuni* (Giallourou et al., 2018) and *Shigella flexneri* (Medeiros PH et al., 2019) resulting in diarrhea in C57BL/6 mice. This current study also used the same antibiotic cocktail to enable the assessment of disease outcomes associated with EPEC infection. We therefore, describe a weaned murine model in which the microbiota have been disrupted *via* broad-spectrum antibiotics to enable efficient colonization and clinical outcomes of EPEC infection in mice resulting in growth impairment, diarrhea, intestinal damage, metabolic alterations, and increased intestinal permeability.

## Materials and Methods

### Ethics Statement

The mice used in the study have been handled with strict accordance with the recommendations in the Guide for the Care and Use of Laboratory Animals of the National Institutes of Health. The protocol has been approved by the Committee on the Ethics of Animal Experiments of the University of Virginia (Protocol Number: 3315). All efforts were made to minimize suffering. This is also in accordance with the Institutional Animal Care and Use Committee policies of the University of Virginia. The University of Virginia is accredited by the Association for the Assessment and Accreditation of Laboratory Animal Care, International (AAALAC).

### Mice

Mice used in this study were male, 22 days old, C57BL/6 strain, ordered from Jackson Laboratories (Bar Harbor, ME). Mice weighed approximately 10 grams on arrival and were co-housed in groups of up to 4 animals per cage. The vivarium was kept at a temperature of between 20 and 23°C with a 14-h light and 10-h dark cycle. Mice were allowed to acclimate for 3 days upon arrival. Mice were fed standard rodent house chow diet from arrival and throughout the infection challenge.

### EPEC Inoculum Preparation

Bacterial strains used included: wild type EPEC E2348/69 [(serotype O127:H6) first isolated in 1969 during infantile diarrhea outbreak in Taunton, United Kingdom, it belongs to phylogroup B2 with full length chromosomal nucleotide (Accession number: FM180568)] (Levine et al., 1985; Iguchi et al., 2009) and EPEC E2348/69 Δ*escN* CVD425 (Jarvis et al., 1995). Bacterial cultures were prepared from glycerol stocks maintained at -80°C. Cultures were grown in 20 ml Dulbecco’s modified Eagle’s medium containing phenol red (DMEM) at 37°C in a shaking incubator until cultures turned orange indicating optimal growth, OD_600_ ~ 0.6. Cultures were centrifuged at 3500 x g for 10 min at 4°C. The bacterial pellet was resuspended in DMEM high glucose in order to obtain 10^10^ CFU/ml.

### Enteropathogenic *Escherichia coli* Infection Model

Four days prior to challenge with EPEC, mice were given an antibiotic cocktail of gentamicin (35 mg/L), vancomycin (45 mg/L), metronidazole (215 mg/L), and colistin (850 U/ml) in drinking water for 3 days in order to disrupt resident microbiota, followed by 1 day on normal water in order to clear antibiotics (Bolick et al., 2018). Then, mice were administered 100 µl of 10^10^ CFU/ml (10^10^ bacteria per mouse) bacterial culture in DMEM high glucose orally using 22-gauge feeding needles. Uninfected control mice were administered only 100 µl DMEM high glucose.

**Figure 1:**
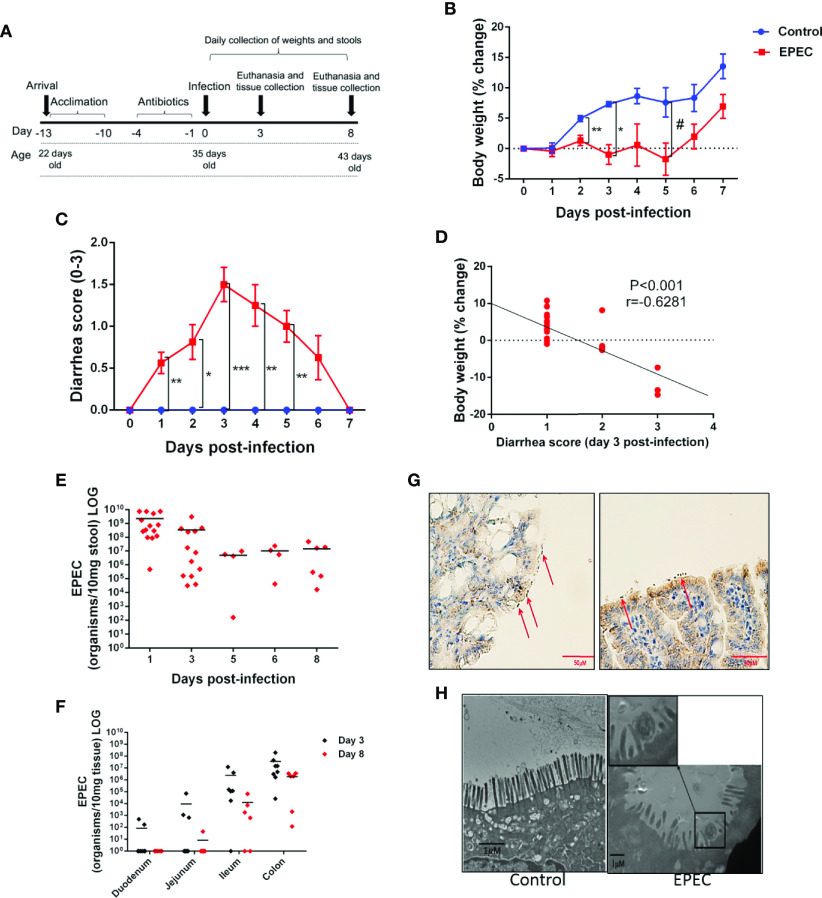
EPEC infection impairs weight gain, induces diarrhea and colon colonization in C57BL/6 mice. **(A)** Experimental timeline of EPEC infection model. Weaned C57BL/6 mice pretreated with antibiotic cocktail were orally infected with EPEC (10^10^ CFU). **(B)** Change in body weight of C57BL/6 mice infected with EPEC (EPEC) or uninfected (Control) (n=12/group). Line graphs represents mean ± SEM. **p< 0.009, *p<0.001 and ^#^*p*< 0.04 using multiple Student’s *t-test*. **(C)** Diarrhea score of EPEC and control mice. Line graphs represent median ± SEM. *p<0.01, **p<0.006 and ***p<0.0001 using Kruskal-Wallis test followed by Dunn’s test. **(D)** Significant negative correlation between diarrhea score and change in body weight at day 3 p.i (Spearman rank test). **(E)**. Quantification of EPEC shedding in stools. **(F)** Quantification of EPEC tissue burden in the intestinal tissues (duodenum, jejunum, ileum and colon) at days 3 and 8 p.i. **(G)** Immunohistochemistry staining of intimin (red arrows), in ileal tissue of EPEC infected mice at day 3 p.i. **(H)** TEM at day 3 p.i., showing disruption of the microvilli of mice infected with EPEC.

After infection, all mice were weighed and stools were collected daily until 8-days post infection (p.i.). Mice were euthanized on days 3, 7, and 8 p.i. **Figure 1A** shows the schematic presentation summarizing the experimental procedure.

### Analysis of Clinical Outcomes

The clinical outcomes, body weight and diarrhea were assessed daily. Body weight was measured for 9 days (starting before infection, day 0) and percentage of changes in body weight was measured based on each individual mouse weight from day 0 before infection. Diarrhea scores were measured until day 7 p.i. Diarrhea score were based on the following 0–4: 0-well-formed pellets; 1- stick stools adhering in microtubes wall; 2-pasty stools with or without mucus; 3-watery stools with or without mucus; and 4-Stools with blood.

### Tissue Burden and Stool Shedding

For stool shedding, DNA was extracted from stools using the QIAamp DNA Stool Mini kit (Qiagen) according to the manufacturer’s instructions. For tissue burden, tissues were homogenized using the beat-beater and DNA was extracted using DNeasy Kit (Qiagen) according to the manufacturer’s instructions. The *eae* (intimin) gene was used as a specific target for detecting EPEC in stools and tissues. Primer sequences included *eae* 5’-CCCGAATTCGGCACAAGCATAAGC-3’ (sense) and 5’-CCCGGATCCGTCTCGCCAGTATTCG-3’ (antisense) (Zhang et al., 2002). Real-time PCR was performed using Bio-Rad CFX under the following conditions: 95°C for 3 min, followed by 40 cycles of 15 s at 95°C, 60 s at 55°C, and lastly 20 s at 72°C.

### EPEC Adherence on the Intestine

Ileal tissue segments from EPEC-infected mice on day 3 p.i. were fixed in 4% formalin, embedded in paraffin, the slides were stained with rabbit anti-intimin at the University of Virginia Histology core, and viewed using light microscope.

### Transmission Electron Microscopy

For TEM, the ileal tissues from EPEC-infected mice on day 3 p.i. and uninfected (control) were fixed with 4% glutaraldehyde. The samples were washed with 1X cacodylate buffer for 10 min and placed in 2% osmium tetroxide for 1 h. Then washed for 10 min with cacodylate buffer and distilled water. Followed by dehydration with 30% ethanol for 10 min and concentrations of 50%, 70%, 95%, and 100% ethanol all for 10 min each. About 1:1 ethanol/propylene oxide was used for 10 min followed by 100% propylene oxide (PO) for 10 min. The samples were then placed in 1:1 of PO/epoxy resin (EPON) overnight followed by 1:2 PO/EPON for 2 h, then 1:4 PO/EPON for 4 h, and lastly 100% EPON overnight. The samples were then embedded in fresh 100% EPON and allowed to bake in a 65°C oven. Ultra-thin sections were cut at 75 nm and picked up on 200 mesh copper grids. Sections were stained with 0.25% lead citrate and 2% uranyl acetate. The slides were viewed using JEOL 1230 microscope, with 4k x 4k CCD camera.

### Histology Analysis

Mice intestinal samples (ileum and colon) from day 3 or day 7 p.i. were fixed in 10% neutral buffered formalin for 20 h, dehydrated and embedded in paraffin. Ileal and colonic sections (5 µm) were stained with hematoxylin and eosin staining (H&E) and were examined using light microscopy. Histopathological scores were performed by a blinded investigator, using previously described methods (Wong et al., 2015). Histological damage scores were determined by quantifying the intensity of epithelial tissue damage (0–3, 0-no damage, 1-mild, 2-moderate, 3-extensive), edema in submucosa layer (0-3), and neutrophil infiltration (0-3).

### Intestinal Inflammatory Response by Enzyme-Linked Immunosorbent Assay

Protein lysates were extracted from the stools, ileum, colon and cecal contents using radioimmunoprecipitation assay (RIPA) buffer (20 mM Tris [pH 7.5], 150 mM NaCl, 1% Nonidet P-40, 0.5% sodium deoxycholate, 1 mM EDTA, 0.1% SDS) containing protease inhibitor cocktail (Roche) and phosphatase inhibitors (1 mM sodium orthovanadate, 5 mM sodium fluoride, 1 mM microcystin LR, and 5 mM β-glycerophosphate). Lysates were centrifuged at 13,000 rpm for 15 min and the supernatant was used to perform the protein assay using the bicinchoninic acid assay (Thermo Fisher Scientific). Inflammatory biomarkers (LCN-2, MPO, IL-23, IL-22, IL-17, GMCSF, IL-33, and IL-10) were measured using a commercial ELISA kit (R&D Systems) according to the manufacturer instructions. Interleukin-6 (IL-6), IL-1β, interferon gamma (INF-γ), TNF-α, KC (analogue to human IL-8), and IL-18 and CRP levels were measured using a ProcartPlex multiplex immunoassay (Invitrogen) by Luminex (Biorad). Biomarkers levels were measured as picograms per milligram of protein.

### Phosphorylated-Signal Transducer *a*nd Activator *o*f Transcription*-*3, Phosphorylated-Cyclic AMP Response Element-Binding Protein and Cleaved Caspase-3 Measurement

Colonic samples from EPEC-infected and control mice collected at day 3 p.i. were homogenized in ice-cold using RIPA buffer containing protease and phosphatase inhibitors. The colonic levels of phosphorylated STAT3 3 (pSTAT3), phosphorylated CREB (pCREB) and cleaved caspase-3 were measured using ELISA kits (R&D Systems) according to the manufacturer’s instructions.

### TaqMan- Real Time Polymerase Chain Reaction

The isolation of total RNA from colon tissues of EPEC-infected (presenting moderate or severe diarrhea) and control mice were performed by using a Qiagen RNeasy mini kit and QIAcube. cDNA was synthetized from 1µg of total RNA, quantified by Qubit 3 fluorometer 3000 (Invitrogen) and purified by deoxyribonuclease I (Invitrogen) treatment, with the iScript cDNA (Bio-Rad) as described by manufacturer instructions. qPCR was performed with 50 ng of cDNA in each well and SensiFAST probe no-ROX mix (Bioline) using a CFX Connect system (Bio-Rad) with the following conditions: 95°C for 2 min, 40 cycles of 95°C for 10 s and 60°C for 50 s. A pre-designed TaqMan array mouse immune fast 96-well plates (Applied Biosystems) was used to assess the expression of 92 genes listed in **Supplementary Table 1**. Glyceraldehyde-3-phosphate dehydrogenase (GAPDH) was used as a reference gene. All fold changes were determined using the ΔΔC_t_ method (Livak and Schmittgen, 2001).

### *In Vivo* Intestinal Permeability Assay

For assessing *in vivo* intestinal permeability fluorescein isothiocyanate (FITC)-labeled dextran assay (4kDa, Sigma Aldrich) was used. Mice were deprived food, with free access to water, for 4 h. Then, 200 µl of FITC-dextran solution (80 mg/ml in water) was administered by oral gavage for each mouse. After 4 h of FITC administration, mice were anesthetized to collect blood using cardiac puncture. Then, the blood samples were centrifuged (5 min, 8,000 rpm, 4°C) and plasma was obtained. Fluorescence intensity in 100 µl of plasma placed on Qubit 0.5 ml-microtubes (Life Technologies) was measured using Qubit 3fluorometer (Life Technologies) using an excitation wavelength of 470 nm. A plasma sample from mice not receiving FITC-dextran solution was used as a blank.

### ^1^H Nuclear Magnetic Resonance Spectroscopy*-*Based Metabolic Profiling

Urine specimen were collected in a sterile 1.5 ml eppendorf tube and placed at -80°C until further analysis. The metabolic profiling was performed on all urine samples using ^1^H nuclear magnetic resonance (NMR) spectroscopy. A 30 µl urine aliquot was combined with 30 µl of phosphate buffer (pH 7.4, 100% D_2_O, 0.2 M Na_2_HPO_4_/NaH_2_PO_4_) containing 1 mM of the internal standard, 3-(trimethylsilyl)-[2,2,3,3^-2^H_4_]-propionic acid (TSP), and 2 mM sodium azide (NaN_3_) as a bacteriocide. Samples were vortexed and spun at 13,000 x g for 10 min and 50 µl of the supernatant was then transferred to 1.7 mm NMR tubes. Spectroscopic analysis was performed on a 600 MHz Bruker Avance™ NMR spectrometer at 300 K using a Bruker BBI probe and an automated SampleJet for tube handling (Bruker, Germany). ^1^H NMR spectra of the urine samples were acquired using a standard one-dimensional pulse sequence [recycle delay (RD) -90°-t_1_–90°-t_m_-90°-acquire free induction decay (FID)]. The water signal was suppressed through irradiation during the RD of 4 s and a mixing time of (t_m_) 100 ms was used. For each spectrum, 64 scans were obtained into 64 K data points using a spectral width of 12.001 ppm. The NMR spectra were calibrated to the TSP resonance at 0 ppm using TopSpin 3.5 NMR software (Bruker, Germany) and imported into MATLAB (R2018a, Mathworks Inc, Natwick, MA) using in-house scripts. Regions containing the TSP, water and urea resonances were removed from the urinary spectra. ^1^H NMR spectra were manually aligned and normalized to the unit area.

### Western Blotting

Colon tissues from EPEC-infected and control mice at day 7 p.i. were collected, lysed using RIPA lysis buffer containing complete EDTA-free protease inhibitor cocktail (Roche) and phosStop (Roche) and centrifuged (17 min, 4°C, 13,000 rpm). Then the supernatant was collected for extracting protein. Protein concentrations were determined through the bicinchoninic acid assay according to the manufacturer`s protocol (Thermo Fisher Scientific). Reduced 60 µg protein samples (previously prepared with sample reducing agent- Invitrogen- and protein loading buffer-LI-COR) were denatured at 95°C for 5 min, separated on NuPAGE 4%–12% BIS-Tris gel (Invitrogen) and transferred to nitrocellulose membranes (Life Technologies) for 2 h. The membranes were then immersed in iBind fluorescent detection solution (Life technologies) and placed in a iBIND automated Western device (Life Technologies) overnight at 4°C for blocking, incubating with primary antibodies (rabbit anti-β-actin, 1:1000, Thermo Fisher Scientific, PA1-183; mouse anti-claudin-2, 3:500, Invitrogen, 325600; rabbit anti-claudin-1, 1:1000, Novus biological, NBP1-77036) and secondary antibodies (Cy3-conjugated AffiniPure donkey anti-rabbit, 711-165-152, 1:1000, Jackson Immuno-Research and Cy5-conjugated AffiniPure donkey anti-mouse, 715-175-150, 1:1000, Jackson ImmunoResearch). Then, the membranes were immersed in ultrapure water and fluorescent signal was detected using the Typhoon system (GE healthcare). Densitometric quantification of bands was performed using ImageJ software (NIH, Bethesda, MD, USA).

### Systemic Inflammation Analysis

Blood collected at day 3 p.i. was centrifuged at 8,000 rpm for 5 min at 4°C in order to obtain the plasma, to measure the levels of SAA as a marker of systemic inflammation. The levels of SAA were measured using a commercial ELISA kit (R&D Systems) according to the manufacturer’s instructions. The results were expressed as picograms per milliliter.

### Statistical Analysis

All data were analyzed using GraphPad Prism 7 software (GraphPad Software). Data are presented as the mean ± standard error of the mean (SEM) or as medians when appropriate. Student’s t test and one-way Analysis of Variance (ANOVA) followed by Tukey’s *test* were used to compare means, and the Kruskal–Wallis and Dunn tests were used to compare medians. Spearman rank test was used to correlation analyses. Differences were considered significant when *p*< 0.05. Experiments were repeated at least two times.

For metabolomics data analysis multivariate statistical modelling was performed including principal component analysis (PCA) using the Imperial Metabolic Profiling and Chemometrics Toolbox (https://github.com/csmsoftware/IMPaCTS) in MATLAB (Version 2018a, Mathworks Inc) and unsupervised hierarchical clustering analysis (HCA) to unveil metabolic differences between EPEC infected and control mice. Unsupervised clustering for all samples was done with the use of the normalized abundance of metabolites that were identified through the PCA models. Hierarchical clusters were generated with the use of an average-linkage method by means of the pdist and linkage functions in the MATLAB bioinformatics toolbox. Heat maps and dendrograms after the HCA were generated with MATLAB imagesc and dendrogram functions, respectively. Pathway analysis was performed using the MetaboAnalyst 4.0 platform (https://www.metaboanalyst.ca/). The raw data supporting the conclusions of this article will be made available by the authors, without undue reservation.

## Results

### EPEC Infection Leads to Growth Impairment and Diarrhea

Depletion of intestinal microbiota by antibiotics has been shown to be effective in promoting colonization by bacterial pathogens (Bolick et al., 2018; Giallourou et al., 2018; Medeiros PH et al., 2019). We therefore, tested whether pretreatment with antibiotics could enable the study of body weight and diarrhea in mice infected with EPEC (10^10^ CFU) (**Figure 1A**). EPEC inhibited the growth of mice when compared to the control group from days 2 to 5 post infection (p.i.) (*p*<0.05, **Figure 1B**). EPEC infection also induced moderate (days 1, 2, and 6 p.i.) to severe diarrhea at days 3 to 5 p.i. (*p*<0.0001, *p*<0.006) (**Figure 1C**). The changes in body weight exhibited by individual EPEC-infected mice were correlated with their individual diarrhea scores at the overall peak of diarrhea and weight decrements on day 3 (**Figure 1D**), showing that higher diarrhea scores tended to associate with greater weight shortfalls.

### EPEC Colonizes the Ileum and Colon in Mice

In order to confirm that growth impairment and diarrhea were promoted by EPEC infection, DNA was extracted from stools of EPEC-infected mice and quantitative PCR was used to measure shedding. As shown in **Figure 1E**, most of the EPEC-infected mice exhibited 10^8^-10^10^ organisms/10 mg stool at day 1 p.i. and less shedding was observed from days 5 to 8 p.i.

Given that EPEC is an intestinal pathogen, we further investigated which intestinal sections were predominantly colonized by EPEC using quantitative PCR to measure tissue burdens. EPEC was found to predominantly colonize the ileum (<10^7^ organisms/mg tissue) and colon (<10^8^ organisms/mg tissue) of mice at day 3 pi (**Figure 1F**), and the same trend was also observed at day 8 p.i. **Figure 1G** shows intimin-stained EPEC adherence on blunted ileal mucosa, with disruption of the microvilli shown by TEM (**Figure 1H**).

These findings indicate that EPEC infection promotes a self-limited symptomatic acute disease in antibiotic-pretreated mice. Fecal shedding of EPEC, and tissue burdens were detected up to day 8 p.i. in infected mice.

### EPEC Infection Promotes Acute Intestinal Tissue Damage and Inflammation

**Figure 2:**
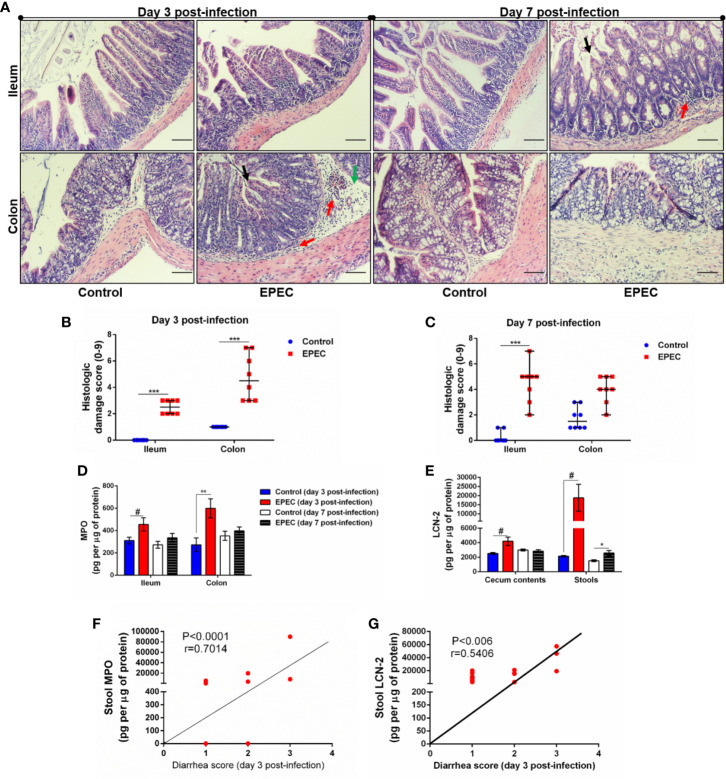
EPEC infection model induces acute colonic damage and inflammation followed by a positive correlation between diarrhea and inflammation markers in stools. **(A)** Representative H&E stains of ileal and colonic tissue collected from control and EPEC infected mice at days 3 and 7 p.i. Scale bars, 100 μm. **(B, C)** Histologic damage score based on epithelial damage (black arrow), mucosal edema (green arrow) and inflammatory cell infiltrate (red arrow) in the ileal and colonic tissue of uninfected (control group) and EPEC-infected (EPEC group) **(B)** mice at day 3 and **(C)** mice at day 7 p.i. Bars represent median ± SEM (n=8). ***p<0.0001 using Kruskal-Wallis test followed by Dunn’s test. **(D)** MPO levels measured in intestinal tissue lysates (ileum and colon) collected at days 3 and 7 p.i. **(E)** LCN-2 levels measured in the cecal and stool lysates collected at days 3 and 7 p.i. Bars represent mean ± SEM (n=8). ^#^*p*<0.05, *p<0.03 and **p<0.007 using multiple Student’s *t-test*. **(F)** Significant positive correlation between diarrhea score and MPO levels in stools of EPEC-infected mice at day 3 p.i (Spearman rank test). **(G)** Significant positive correlation between diarrhea score and LCN-2 levels in stools of EPEC-infected mice at day 3 p.i (Spearman rank test).

Given that our EPEC infection model resulted in a significant colonization in the ileum and colon, we next investigated whether EPEC infection promotes ileal and colonic damage. The ileal and colonic histological damage in EPEC-infected mice was characterized by the loss of epithelial integrity, moderate edema in the submucosa and infiltration of inflammatory cells into the lamina propria and submucosa, with significant histology score differences from controls in both ileum and colon at day 3, and persistent significant differences in the ileum extending to day 7. The damage was found to be greater in colon compared to control mice (**Figure 2A**) at day 3 p.i., as confirmed by measurement of histologic damage score (*p*<0.0001, **Figure 2B**). On day 7 p.i., the damage in the ileum of EPEC-infected mice was higher when compared to the control mice (*p*<0.0001, **Figures 2A, C**).

Myeloperoxidase (MPO), a marker of neutrophil activity in intestinal mucosa, and lipocalin-2 (LCN-2), a glycoprotein upregulated in tissue damage under infection conditions, have been considered as biomarkers of environmental enteric dysfunction, including EPEC infection in children (Prata et al., 2016; Kosek et al., 2017). To ensure that our EPEC infection model mimics the alterations of these biomarkers as observed in children, we measured MPO and LCN-2 in the ileal and colonic tissues, as well as in cecal contents and stools. We found increased MPO levels in ileum and colon tissues of EPEC-infected mice at day 3 p.i. when compared to the control group (*p*<0.05 and *p*<0.03 respectively, **Figure 2D**). Of note, a trend of increase in MPO levels was also observed in the cecal contents and stools at day 3p.i. (**Supplementary Figure 1**), however no statistical significance was found. On day 7 p.i., MPO levels were reduced in the intestinal (ileum and colon), cecal contents and stools of EPEC infected mice compared to controls (**Figure 2D**, **Supplementary Figure 1**). However, increased LCN-2 levels were found in cecal contents (day 3 p.i.) and stools (days 3 and 7 p.i.) of EPEC-infected mice when compared to control mice (*p*<0.05-day 3 p.i. or *p*<0.03-day 7 p.i., **Figure 2E**).

We found that the diarrhea score correlated with MPO or LCN-2 levels in stools samples of EPEC-infected mice at day 3 p.i., when mice exhibited higher diarrhea score, a positive correlation was found between diarrhea score and MPO levels in stools (*p*<0.0001, r=0.7014, **Figure 2F**). Positive correlation was also seen of diarrhea score with LCN-2 levels at day 3 p.i. (*p*<0.006, r=0.5406, **Figure 2G**). These data indicated that high diarrhea scores are associated with increased MPO and LCN-2 levels.

### EPEC Infection Alters Pro-Inflammatory and Anti-Inflammatory Cytokine Synthesis in Ileum and Colon in a Stage Disease-Dependent Manner in Mice

**Figure 3:**
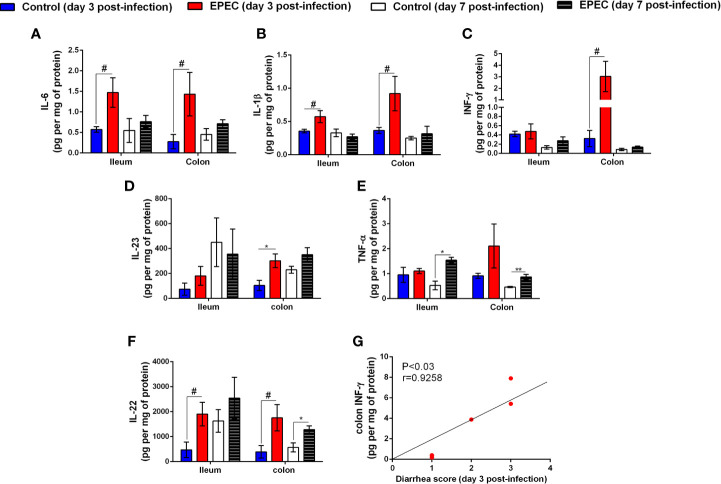
EPEC infection increases pro-inflammatory mediators, and diarrhea scores correlates positively with INF-γ and KC levels in colonic tissues of EPEC infected mice. **(A)** Levels of IL-6, **(B)** IL-1β, **(C)** INF-γ, **(D)** IL-23, **(E)** TNF-α, and **(F)** IL-22 in the ileal and colonic tissues of control and EPEC infected mice at days 3 and 7 p.i. were measured by ELISA. Bars represent mean ± SEM (n=8). ^#^*p*<0.05, *p<0.01 and **p<0.001 using multiple Student’s *t-test*. **(G)** Significant positive correlation between diarrhea score and INF-γ levels in colonic tissues of EPEC infected mice at day 3 p.i (Spearman rank test).

Next, we further analyzed which pro-inflammatory [IL-6, IL-1β, INF-γ, IL-23, tumor necrosis factor-α (TNF-α), IL-17, IL-18] and anti-inflammatory (IL-22, IL-33, IL-10) cytokines were affected by EPEC infection during acute (day 3 p.i.) and later (day 7 p.i.) stage of the disease. As shown in **Figure 3**, higher levels of IL-6 (*p*<0.05), IL-1β (*p*<0.05), INF-γ (*p*<0.05), IL-23 (*p*<0.01), and IL-22 (*p*<0.05) were detected in colonic tissues of EPEC-infected mice at day 3 p.i. and were significantly different when compared to controls (**Figures 3A–D, F**). EPEC infection in the ileal tissue resulted in significant increase in IL-6, IL-1β, and IL-22 at day 3 p.i. when compared to control mice (*p<*0.05, **Figures 3A, B, F**). In the later stage of disease (day 7 p.i.), TNF-α (ileal and colonic tissues) and IL-22 (colonic tissues) were significantly increased in EPEC-infected mice when compared to control mice (*p*<0.01, **Figures 3E, G**). In addition, increase of KC in the ileal and colonic tissues was observed, however, not statistically significant (**Figure 3F**).

We analyzed the correlation of diarrhea severity with the levels of INF-γ in colonic tissues of EPEC-infected mice and found a strong positive correlation between diarrhea score and colonic INF-γ levels (*p*<0.03, r=0.9258, **Figure 3H**). We did not find any change in the levels of IL-17, IL-18, granulocyte-macrophage colony–stimulating factor (GMCSF), IL-33, and IL-10 in ileal and colonic tissues samples of EPEC-infected mice when compared to the controls (**Supplementary Figures 2A–E**).

Taken together, these data indicate that EPEC infection promotes two-phase of disease in mice; an acute stage characterized by growth impairment, intestinal damage, presence of diarrhea and increased of MPO, LCN-2, IL-6, IL-1β, INF-γ, IL-23, and IL-22; and a later stage with an increase in LCN-2 and TNF-α levels in an absence of diarrhea. In addition, the data suggests that the colon is more affected by EPEC infection in mice.

### EPEC-Infected Mice With Diarrhea Demonstrates Upregulation of Pro-Inflammatory Cytokines, Inflammatory Markers, STAT, and Apoptosis Markers in Colon

**Figure 4:**
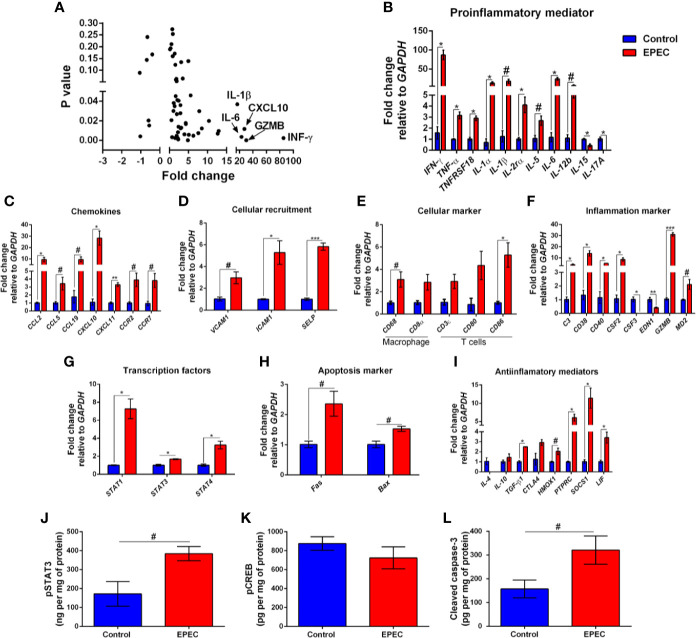
EPEC-infected mice with soft or unformed stools exhibit higher expression of mRNA for pro-inflammatory mediators, chemokines, and other inflammatory markers. **(A)** mRNA expression fold changes (on the x axis) and the corresponding *p*-values (on the y axis). Gene names are labeled next to highlighted significant results. Taqman qPCR analysis of **(B)** pro-inflammatory, **(C)** chemokines, **(D)** cellular recruitment, **(E)** cellular marker, **(F)** inflammation marker, **(G)** transcription factors, **(H)** apoptosis marker, and **(I)** anti-inflammatory genes in the colonic tissues of control and EPEC-infected mice with moderate (score=2) or severe (score=3) diarrhea scores at day 3 p.i. **(A–I)** Expression levels were normalized with GAPDH, as an internal housekeeping gene. Bars represent mean ± SEM (n=3). ^#^*p*<0.05, *p<0.01, **p<0.001 and ***p<0.0001 using multiple Student’s *t-test*. Levels of **(J)** pSTAT3, **(K)** pCREB and **(L)** cleaved caspase-3 in colonic tissue lysates of control and EPEC-infected mice at day 3 p.i. measured using ELISA. Bars represent mean ± SEM (n=8). ^#^*p*<0.05 using Student’s *t-test*.

Due to an increase in diarrhea severity and colonic INF-γ levels that were positively correlated on day 3 p.i., we evaluated the profile of gene expression from the colonic tissues of EPEC-infected mice with diarrhea and controls using Taqman assay. In total, 92 genes were evaluated, among these 37 were upregulated and four were downregulated (*p*<0.05, **Figure 4A**). *INF-γ*, *GZMB*, *CXCL10*, *IL-6*, and *IL-1β* were the most upregulated genes, showing approximately 85, 30, 27, 23, and 16-fold-change in relation to controls (*p*<0.05, **Figure 4A**). In mice with diarrhea, EPEC infection resulted in upregulation of pro-inflammatory mediators (*INF-γ, TNF-α, TNFRS, IL-1α, IL-1β, IL-2Rα, IL-5, IL-6, and IL-12b*, **Figure 4B**), chemokines (*CCL2, CCL5, CCL19, CXCL10*, and *CXCL11*, **Figure 4C**), chemokine receptors (*CCR2* and *CCR7*, **Figure 4C**), cellular recruitment (*VCAM1*, *ICAM1*, and *SELP*, **Figure 4D**), *CD68* (a macrophage marker, **Figure 4E**), *CD3ϵ* (marker of T-cell activation, **Figure 4E**), inflammation markers (*C3*, *CD38*, *CD40*, *CSF2*, *GZMB*, and *MD2*, **Figure 4F**), transcription factors [phosphorylated-signal transducer and activator of transcription-1 (*STAT1*), *STAT3*, and *STAT4*, **Figure 4G**] and apoptosis markers (*Fas* and *Bax*, **Figure 4H**) in colon of mice at day 3 p.i. In addition, EPEC-infection also upregulated gene expression of anti-inflammatory mediators, such as *TGF-β1*, *HMOX1*, *PTPRC*, *SOCS1*, and *LIF* when compared to control mice (*p*<0.05, **Figure 4I**).

Signal transducer and activator of transcription 3 (STAT3) is a transcription factor involved in response of cytokines such as IL-6, and its activation results in expression of target genes involved in inflammatory and anti-inflammatory responses (Griesinger et al., 2015; Aden et al., 2016). To investigate the levels of phosphorylated STAT3 (pSTAT3), its active form, we found increased levels of pSTAT3 in the colon of mice infected with EPEC on day 3 p.i. (*p*<0.05, **Figure 4J**). Cyclic AMP Response Element-Binding protein (CREB) is another transcription factor involved on the transcription of inflammatory (such as IL-6, IL-2, and TNF-α) and anti-inflammatory (IL-4, IL-10 and IL-13) mediators (Westbom et al., 2014; Zhang et al., 2019). To determine the levels of phosphorylated CREB (pCREB), its active form, we examined, but did not find a difference between EPEC-infected mice and controls on day 3 p.i. (**Figure 4K**).

Given that EPEC infection increased the gene expression of apoptosis markers, we further evaluated the levels of cleaved caspase-3 using ELISA. We found that EPEC infection induced an increase in cleaved caspase-3 in the colonic tissues when compared to control mice at day 3 p.i. (*p*<0.05, **Figure 4L**), confirming an apoptosis process during EPEC infection.

### EPEC Infection Model Induces Metabolic Perturbations

**Figure 5:**
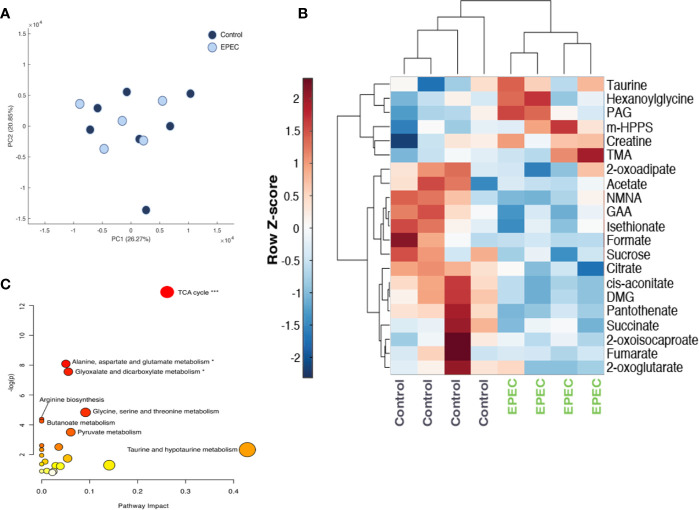
Metabolomic analysis of urinary specimens collected from age-matched, control, and EPEC infected mice. **(A)** Principal component analysis **(**PCA) scores plot at day 1 p.i. showing no difference in the metabolic profiles of between infected and uninfected mice (control n=7, EPEC n=5). **(B)** Unsupervised hierarchical clustering and heat-map of urinary metabolites from control and EPEC-infected mice at day 3 p.i (n=4). Each row represents a metabolite and each column represents a mouse sample. The row Z-score (scaled expression value) of each metabolite is plotted in red-blue color. The red color indicates metabolites, which are high in abundance and blue indicates metabolites low in abundance. DMG, dimethylglycine; GAA, guanidinoacetic acid; m-HPPS, m-hydroxyphenylpropionylsulfate; NMNA, N-methyl-nicotinic acid; PAG, phenylacetylglycine; TMA, trimethylamine. **(C)** Pathway analysis plot created using MetaboAnalyst 4.0 showing metabolic pathway alterations induced by EPEC. Each pathway is represented as a circle. Darker colors indicate more significant changes of metabolites annotated on the corresponding pathway. The x-axis represents the pathway impact value computed from pathway topological analysis, and the y-axis is the-log of the *p*-value obtained from pathway enrichment analysis. The pathways that were most significantly changed are characterized by both a high-log(*p*) value and high impact value (top right region). FDR adjusted *p-*values, *p<0.05, ***p<0.0001.

Metabolic perturbations following EPEC infection were further analyzed using Principal Component analysis (PCA). Urinary metabolic profiles of each of the mice infected with EPEC were compared to the age-matched uninfected mice at days 1 and 3 p.i. No differences were observed between the controls and EPEC infected mice on the day 1 p.i. (**Figure 5A**).

EPEC infection-driven metabolic variation was, however, observed at day 3 pi (PCA model: Q^2^ = 0.25, R^2^ = 0.59 (1000 permutations). EPEC infection resulted in reduced excretion of TCA cycle metabolites (succinate, cis-aconitate, citrate, 2-oxoglutarate and fumarate) and choline related metabolite, dimethylglycine (DMG) and increased trimethylamine (TMA) (**Figure 5B**). Lower urinary excretion of the tryptophan catabolite N-methylnicotinic acid (NMNA), the creatinine precursor guanidinoacetic acid (GAA) and the amino acid catabolites 2-oxoisocaproate, 2-oxoadipate were also observed. Isethionate, formate, pantothenate, and sucrose were also excreted in lower amounts by EPEC infected mice. Increases in the excretion of gut microbial-derived metabolites [acetate, phenylacetylglycine (PAG), m-hydroxyphenylpropionylsulfate (m-HPPS)], were observed in infected mice. Urinary excretion of taurine, creatine, and b-oxidation product hexanoylglycine were also elevated at day 3 p.i (**Figure 5B**).

Pathway analysis revealed that the TCA cycle was the biochemical pathway most influenced by the infection (Impact: 0.26, *p*-value: 2.5E-6, FDR adjusted *p*-value: 2.1E-4) (**Figure 5C**).

### EPEC Infection Increases Intestinal Permeability and Decreases Colonic Claudin-1 Expression in Mice

**Figure 6:**
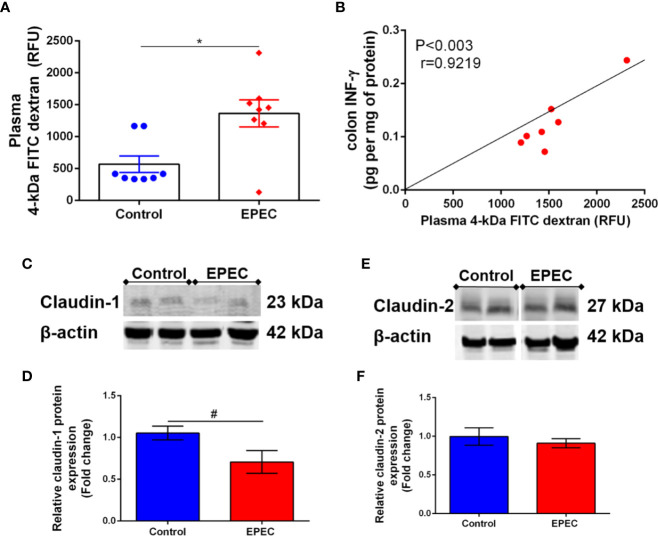
EPEC infection model increases intestinal permeability and decreases claudin-1 expression in colon of mice. **(A)** Intestinal permeability was determined in the plasma of control and EPEC-infected mice at day 7 p.i. using FITC dextran assay. Each point represents a mouse. Lines represent mean ± SEM (n=8). *p < 0.006 using Student’s *t-test*. **(B)** Significant positive correlation between diarrhea score and INF-γ levels in colon tissues of EPEC-infected mice at day 7 p.i (Spearman rank test). **(C)**. Representative western blots of levels of claudin-1 and β-actin in colonic tissues of control and EPEC-infected mice at day 7 p.i. **(D)** Quantification of western blot bands of claudin-1 and β-actin. Bars represent mean ± SEM (n=3). ^#^*p* < 0.03 using Student’s *t-test*. **(E)** Representative western blots of levels of claudin-2 and β-actin in colonic tissues of control and EPEC-infected mice at day 7 p.i. **(F)** Quantification of western blot bands of Claudin-2 and β-actin. Bars represent mean ± SEM (n=3).

Alteration on intestinal permeability related to EPEC infection has been reported in children (Johansen et al., 1989). Given that the intestinal tissues of EPEC-infected mice showed increase in inflammatory markers on later stage of disease, we investigated whether intestinal permeability was altered by using a FITC dextran assay in our experimental model. As shown in **Figure 6A**, EPEC infection resulted in increased levels of plasma 4kDa FITC dextran, indicating higher intestinal permeability when compared to control mice at day 7 p.i. (*p*<0.006). A strong positive correlation was found between the levels of plasma 4kDa FITC dextran and colonic INF-γ at day 7 p.i. in EPEC-infected mice (*p*<0.003, r=0.9219, **Figure 6B**).

Tight junctions play a crucial role in regulating intestinal permeability, we therefore, investigated if EPEC infection alters claudin-1 and claudin-2 expression in the colon of mice, and found that EPEC infection decreased claudin-1 (*p*<0.03, **Figures 6C, D**), but not claudin 2 (**Figures 6E, F**) in the colon when compared to control mice. Uncropped versions of **Figures 6C, E** are available in **Supplementary Data** (**Supplementary Figures 3A, B**).

### Loss of escN Expression in EPEC Inhibits Intestinal and Systemic Inflammation Induced by Wild-Type Enteropathogenic *Escherichia coli* Infection Model Without Changes in Stools Shedding in Mice

**Figure 7:**
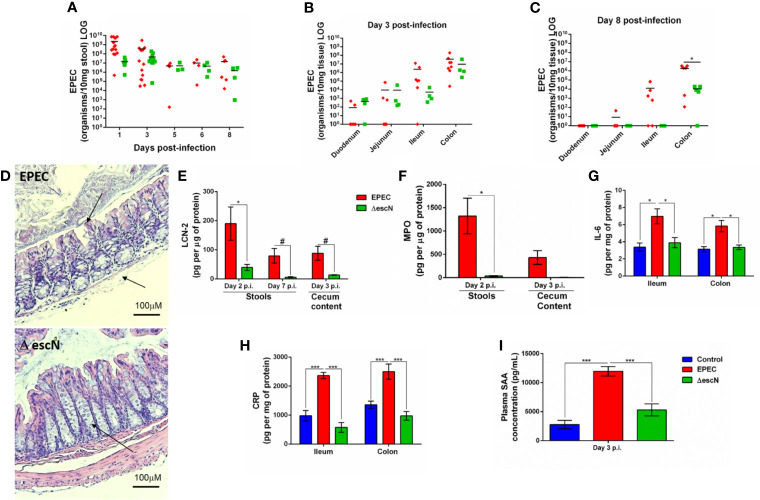
Inactivation of *escN* in EPEC decreases colonization in colon and reduces intestinal and systemic inflammation promoted by EPEC infection in mice. **(A)** Quantification of EPEC shedding in stools of WT EPEC (EPEC) or Δ*escN* EPEC infected mice. **(B, C)** Quantification of EPEC tissue burden in intestinal tissues (duodenum, jejunum, ileum and colon) at day 3 and 8 p.i. Bars represent mean ± SEM. *p < 0.01 using multiple Student’s *t-test*. **(D)** Representative H&E stains of colon from WT EPEC (EPEC) or Δ*escN* infected mice at day 3 p.i. Scale bars, 100 μm. **(E)** LCN-2 levels measured in stools (days 2 and 7 p.i.) and cecal contents (day 3 p.i.) of WT EPEC (EPEC) or Δ*escN* infected mice. **(F)** MPO levels measured in stools (day 2 p.i.) and cecal contents (day 3 p.i.). **(G)** IL-6 levels measured in the ileal and colonic tissue lysates at day 3 p.i. using ELISA **(H)** CRP levels measured in the ileal and colonic tissue lysates of uninfected (control, in blue), WT EPEC (in red) or Δ*escN* EPEC (in green) infected mice at day 3 p.i (**I**). Concentration of SAA in plasma at day 3 p.i. was determined using ELISA. Bars represent mean ± SEM (n=8). ^#^*p* < 0.05, *p < 0.01 and ***p < 0.0001 using multiple Student’s *t-test* **(E, F)** or One-way ANOVA followed by Turkey´s test **(G–I)**.

The T3SS is essential for EPEC pathogenesis, and disruption of the *escN* gene (ATPase energizer), can lead to inefficient injection of EPEC effectors into the host cell (Andrade et al., 2007). We therefore, tested whether inactivation of *escN* in EPEC strain could affect changes in body weight, intestinal and systemic inflammation in our EPEC infection model. The Δ*escN* EPEC-infected mice exhibited weight gain when compared to wild-type (WT) EPEC-infected mice on days 1 and 3 p.i (*p*<0.05 and *p*<0.001, respectively, **Supplementary Figure 4**). Deletion of Δ*escN* did not affect EPEC shedding in the stools (**Figure 7A**) and colonization in the intestinal tissues (**Figure 7B**). However, at day 8 p.i., tissue burden of Δ*escN* EPEC infected mice was detected only in the colonic tissue (*p*<0.01 **Figure 7C**). No histological changes were observed in the colon of mice infected with Δ*escN* EPEC when compared to mice infected with WT EPEC (**Figure 7D**). Furthermore, Δ*escN* EPEC infection resulted in decreased LCN-2 in the stools (days 2 and 7 p.i., *p*<0.01 and *p*<0.05, respectively) and cecal contents (day 3 p.i., *p*<0.05) (**Figure 7E**); and MPO in stools (day 2 p.i., *p*<0.01) (**Figure 7F**), as well as IL-6 in ileum and colon (day 3 p.i., *p*<0.01) (**Figure 7G**).

To assess whether WT EPEC infection induces systemic inflammation, as well as the role of the T3SS using Δ*escN* during infection, C-reactive protein (CRP) in intestinal tissues (ileum and colon) and serum amyloid A (SAA) in plasma of mice collected at day 3 p.i. were measured. WT EPEC infection resulted in increased the levels of CRP in the intestinal tissues (*p*<0.0001, **Figure 7H**) and increased SAA (*p*<0.0001, **Figure 7I**) when compared to control mice. Whereas deletion of Δ*escN* in EPEC led to a significant decrease in levels of these markers of systemic inflammation at day 3 p.i. when compared to WT EPEC-infected mice (*p*<0.0001, **Figures 7H, I**).

Taken together, these findings showed that EPEC induces later colonic colonization, intestinal and systemic inflammation, in an *escN-*encoded T3SS*-*dependent manner, confirming the effectivity of this new EPEC infection model in mice.

## Discussion

Typical EPEC infections have been suggested to be associated with inflammatory enteropathy and/or diarrhea in resource-limited populations (Platts-Mills et al., 2015; Rogawski et al., 2018). Infections caused by EPEC have been widely reported in *in vitro* studies to demonstrate the effects of adherence traits (Knutton et al., 1999) and the T3SS (Andrade et al., 2007). However, there was still a need for a suitable *in vivo* model that clearly shows the effects of human EPEC infection in an intestinal environment. *Citrobacter rodentium* is a natural murine pathogen that has also been shown to cause A/E lesions, with the formation of pedestal structures and polarized actin accumulation at the site of infection analogous to that seen in human EPEC and Enterohemorrhagic *E. coli* (EHEC) infections (Luperchio and Schauer, 2001; Mundy et al., 2006).

Herein an EPEC murine infection model is reported using mice pretreated with antibiotic cocktail (vancomycin, gentamicin, metronidazole and colistin) to enable colonization, and induce growth impairment, acute diarrhea, intestinal damage and inflammation, as well as metabolomic perturbations and intestinal permeability alterations. Efforts to develop an EPEC model that mimics clinical outcomes, such as growth impairment and diarrhea as observed in humans have been a challenge for *in vivo* studies (Johansen et al., 1989; Savkovic et al., 2005; Law et al., 2013; Dupont et al., 2016). We also identified a two-phase disease promoted by the EPEC infection model, an acute symptomatic and a later asymptomatic phase. In the acute symptomatic phase of the disease, EPEC-infected mice had growth impairment and diarrhea as clinical outcomes, and this was accompanied with ileal and colonic damage (loss of integrity of epithelial cells, edema in submucosa and intense infiltrate of inflammatory cells) and intense inflammation. Similar findings showing disruption of colonic damage following EPEC infection has also been previously reported (Zhang et al., 2012).

In humans, EPEC infection promotes watery diarrhea and dehydration (Guerrant et al., 2011). Most of the EPEC infected mice developed moderate to severe diarrhea at day 3 p.i. Previously, C57BL/6 mice infected with EPEC have been reported to develop semi-solid stools in the proximal colon with no apparent diarrhea (Savkovic et al., 2005).

MPO has been used as biomarker of enteropathy in clinical studies (Guerrant et al., 2016; McCormick et al., 2017), also exhibiting inflammation, growth and development decrements in children infected with different enteropathogens in low-income countries. In our present study, MPO was increased during acute phase of disease, in the intestinal tissues (ileum and colon); MPO was detected mainly in stools of mice that developed soft or unformed stools or diarrhea. In addition, LCN-2 which is known as a neutrophil gelatinase-associated protein expressed by intestinal epithelial cells (Makhezer et al., 2019), was detected in higher concentrations in stools of all the mice that were infected with EPEC. Particularly impressive in our model was the striking inflammatory enteropathy (as evidenced by fecal LCN-2 and MPO during acute infection) with EPEC infected mice developing watery mucoid stools.

EPEC infection in infant C57BL/6 mice has been previously reported to colonize the small intestine and colon for 3 days as a result of human milk oligosaccharides administration (Manthey et al., 2014). Here, the ileum and colon were markedly colonized by EPEC during infection challenge. Similar to our findings, EPEC infection in other mouse models have also been reported to colonize the ileal and colonic tissues (Savkovic et al., 2005; Dupont et al., 2016). Moreover, the colon in our model was most affected by EPEC infection with higher colonization by EPEC; and similar high EPEC colonization has been previously reported in the colon of germ-free mice albeit without mention of diarrhea (Dupont et al., 2016).

The pro-inflammatory cytokines such as IL-6, IL-1β, IL-23, IL-22, and INF-γ were increased in colon of mice infected with EPEC. IL-6 is a pleiotropic cytokine showing a pro-inflammatory phenotype and is protective against infection. For instance, deficiency of IL-6 in C57BL/6 mice has been reported to cause colonic damage, increase infiltrate of inflammatory cells and apoptosis during infection with *C. rodentium* (Dann et al., 2008). Mice lacking IL-1β are more susceptible to *C. rodentium-*induced colonic inflammation (Liu et al., 2012), in contrast blocking IL-1β in EPEC-infected mice with persistent IL-1β response decreased the colonic damage (Sham et al., 2013), suggesting the role of IL-1β during intestinal infection in a concentration- and timing-dependent manner. Binding of IL-1β, to IL-1 receptor type I (IL-1RI) and activation of nuclear factor κB (NF-κB), promotes the recruitment of inflammatory cells at the site of inflammation by inducing the expression of adhesion molecules on endothelial cells and the release of chemokines (Bolick et al., 2007; Gabay et al., 2010). In our model, EPEC infection increased the expression of adhesion molecules (VCAM1, ICAM1, and SELP), as well as chemokines (CCL2, CCL5, CCL19, CXCL10, and CXCL11) and the chemokine receptors CCR2 (activated by CCL2 and expressed by macrophage and lymphocytes) and CCR7 (activated by CCL19, promoting migration of dendritic cells, monocytes and T cells) (Pezoldt et al., 2017), contributing to the intense recruitment of inflammatory cells in the colon, but not in the ileum, of EPEC-infected mice.

Similar to IL-6, INF-γ is a pleiotropic protein that promotes the transcription of pro-inflammatory mediators and CXCL10 (by binding to CXCR3 in order to promote the recruitment of monocytes/macrophages and T cells at the site of infection) (Lee et al., 2017), and were both upregulated in the colon of mice following challenge with EPEC, likely activating STAT1 by binding to INF-γ receptor (INFGR) (Green et al., 2017). In fecal samples from children with symptomatically EPEC infection, intermediate levels of INF-γ have been associated with an increase in infection duration (Long et al., 2010). INF-γ levels were increased in colonic tissues in our study, and severity of diarrhea was associated with higher levels of INF-γ in EPEC-infected mice at day 3 p.i.

IL-23, was also increased following EPEC infection, and has been shown to be required to promote IL-22 expression, a cytokine involved in promoting tissue regeneration and regulating inflammation, and also to negatively control the potentially deleterious production of IL-12 (Aychek et al., 2015). The data therefore, suggests that an increase in these cytokines during EPEC-infection in our model is protective, but not enough to prevent the intestinal damage promoted by EPEC. In *C. rodentium* infection, lack of IL-23 in macrophages led to increased mortality in mice (Aychek et al., 2015). Here, we also provided data suggesting that our EPEC infection model was able to activate NF-κB *via* IL-1β, STAT1 *via* INF-γ, and STAT3 *via* IL-22 and IL-6, but not CREB. STAT1 and STAT3 contribute to the expression of pro-apoptotic and anti-apoptotic genes respectively (Wittkopf et al., 2015; Lee et al., 2017). However, in the present study, it seemed that the response mediated by STAT1 (whose expression was higher than STAT3 in colon of EPEC-infected mice) prevailed over anti-apoptotic response promoted by STAT3, once increased cleaved caspase, a marker of apoptosis, were detected. Moreover, because our EPEC infection model exhibits evident diarrhea more investigation of how these cytokines contribute to its pathogeneses is needed.

Furthermore, during acute phase of infection, EPEC infection resulted in perturbations of multiple biochemical pathways, with the TCA cycle intermediates appearing to be the most sensitive to EPEC infection. The TCA cycle in *E. coli* is linked to energy metabolism in which CO_2_ concomitant is oxidized from pyruvate leading to production of NADH and FADH_2_ (Alteri and Mobley, 2012). In our model, the TCA cycle metabolites were excreted in lower quantities following EPEC infection, suggesting that energy production was reduced or conserved in the infected host. A shut-down of the TCA cycle during infection suggests that the energy requirements of the host were not met, potentially explaining in part the significant weight loss in the infected mice. *C. jejuni* infection in zinc deficient mice have also been reported to perturb the TCA cycle, affecting amino acid and muscle catabolism as a result of increased creatine excretion (Giallourou et al., 2018). Pantothenate is the key precursor of the fundamental TCA cycle cofactor, coenzyme A (Leonardi et al.). Reduced pantothenate excretion following EPEC colonization further adds to the TCA cycle disruption by infection. Interestingly, excretion of creatine which is a source for energy production in the form of ATP was also increased during infection. Sugiharto and colleagues reported on post-weaning pigs infected with *E. coli* F18 and found that there was a reduction in creatine and betaine which was due to inhibition of antioxidant system that resulted in piglets developing diarrhea (Sugiharto et al., 2014). Taurine has been shown to possess antioxidant properties and its concentrations are elevated in inflamed tissues where oxidants are abundant (Jeon et al., 2009; Oliveira et al., 2010). EPEC infection in this study was characterized by elevated urinary taurine excretion. As we have previously observed (Swann et al., 2011), treating rodents with antibiotics suppresses the bacterial metabolism of taurine thus increasing taurine bioavailability and uptake in the host reflected by greater urinary taurine excretion. Metabolites derived from bacteria in the gut were excreted in greater amounts following infection suggesting gut microbial metabolism was altered by EPEC infection. These findings help to understand host metabolism during infection, suggesting potential pathways to be further explored and targeted in future studies.

In relation to later phase of EPEC infection, we observed an increase in TNF-α in the colon and ileum, as well as increased LCN-2 in stools samples and increased intestinal permeability and decreased claudin-1. Despite TNF-α gene expression, as well as its receptor TNFRS being increased during the acute phase of disease, the TNF-α protein levels were increased in intestinal tissues of EPEC-infected mice only during the later phase. TNF-α synthesis is promoted by NF-κB activation which in turn can be promoted by IL-1β (Kalliolias and Ivashkiv, 2016). The biological effects of TNF-α mediated by binding to TNFRS include inflammation, apoptosis and tissue regeneration *via* activation of NF-κB, caspase-8 and AKT respectively (Kalliolias and Ivashkiv, 2016). Similar to our findings, later increases of TNF-α in the ileum and colon has been observed by others in EPEC-infected mice at day 5 p.i (Shifflett et al., 2005), findings that we showed at day 7 p.i. This increase in TNF-α was associated with increased LCN-2 in stools, indicating the presence of intestinal damage and inflammation, despite partial recovery from the acute phase of disease. TNF-α has been shown to induce LCN-2 expression by activating NF-κB (Makhezer et al., 2019). TNF-α and INF-γ have been associated with a loss of integrity of the intestinal epithelial barrier (Chelakkot et al., 2018). Despite TNF-α, but not INF-γ, being increased in colonic tissues in EPEC-infected mice, only INF-γ levels were associated positively with an increase in intestinal permeability; a similar association has also been reported in an *in vitro* study using T84 epithelial cell monolayer (Smyth et al., 2011). The permeability of the intestinal barrier is regulated by the tight junction proteins (Wang et al., 2017). EPEC infection has been reported to impair tight junction barrier function of ileal and colonic mucosa (Mundy et al., 2006; Guttman et al., 2006; Zhang et al., 2010; Zhang et al., 2012; Ugalde-Silva et al., 2016). Claudin-1, a component of tight junction expressed by epithelial cells from small and large intestine, is responsible for increasing barrier tightness (Luissint et al., 2016). In our model, a decrease of claudin-1 in the colon of EPEC-infected mice was associated with an increase in intestinal permeability. Although we detected alterations in intestinal permeability at the later stage of EPEC infection, this might have been due in part to an increase in systemic markers (SAA and CRP) that were detected at day 3 p.i. during the acute phase leading to disruption of intestinal tight junctions. SAA has been a biomarker of enteropathy in clinical studies also associated with inflammation, and with growth and developmental impairment in children infected with multiple enteropathogens in low-income countries (Guerrant et al., 2016).

The T3SS is essential for EPEC pathogenesis and requires an effective ATPase energizer, *escN* (Gauthier et al., 2003; Andrade et al., 2007; Zarivach et al., 2007). Here, we also demonstrated that mice infected with *escN* deletion mutant resulted in diminished growth impairment and inflammation. Even without an effective T3SS, *escN* mutant was able to colonize all sections of the intestinal tissue (due to the presence of functional bfp), albeit at much lower levels to day 8 p.i., as shown by our results. These findings reinforce the importance of a functional T3SS in the virulence of EPEC in this model (Frankel et al., 1998; Andrade et al., 2007).

**Figure 8:**
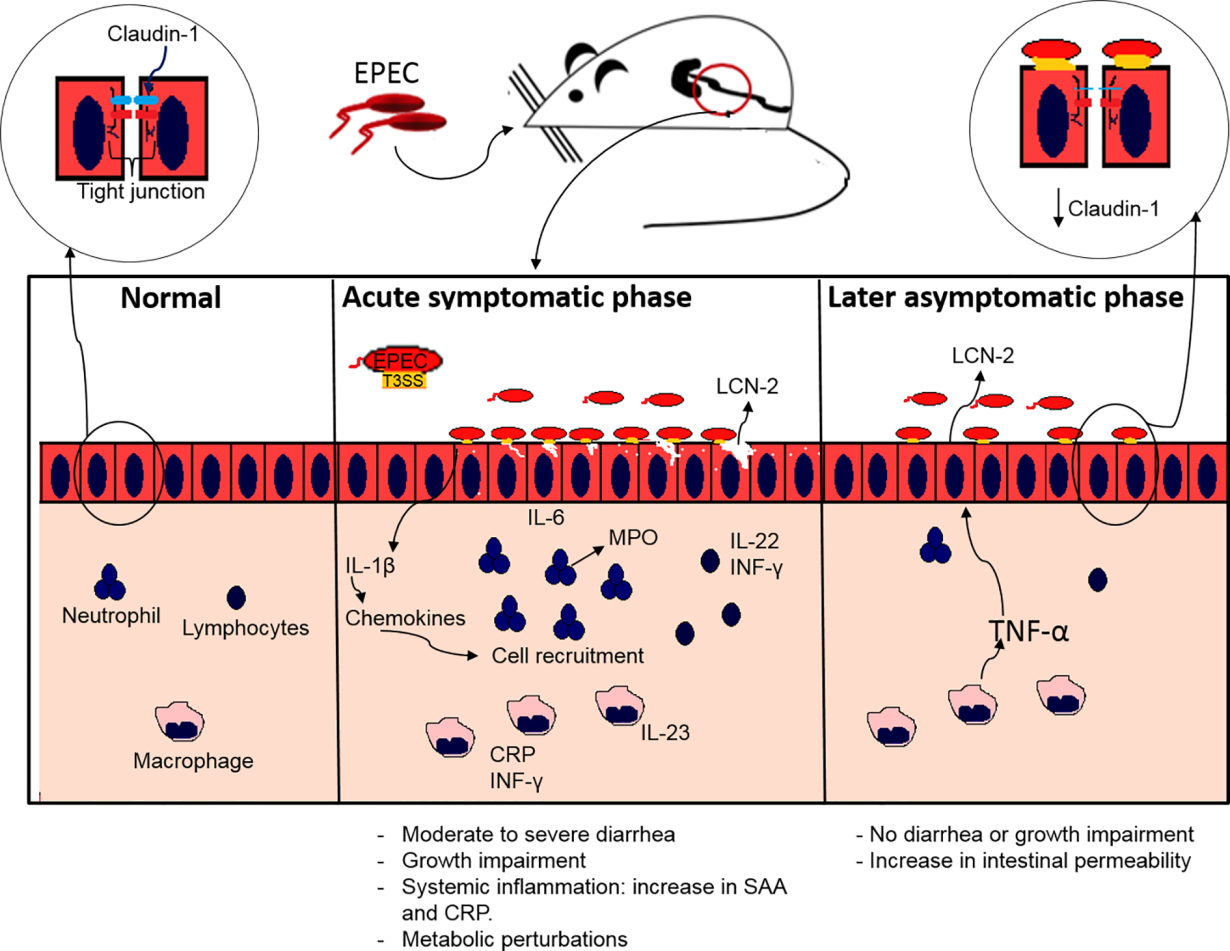
Proposed model of EPEC infection antibiotic pretreated mice displaying acute symptomatic and late asymptomatic phase. During acute symptomatic phase, colonization by EPEC leads to growth impairment, accompanied with moderate to severe diarrhea. Adherence of EPEC on the intestinal epithelial cells leads to increased IL-1β, which in turn stimulates chemokines synthesis, and consequently recruitment of neutrophils, macrophages and lymphocytes, resulting in increased release of MPO by neutrophils and LCN-2 by epithelial cells, as well as pro-inflammatory (IL-6, IL-23, INF-γ) and IL-22 cytokines indicating intestinal inflammation. A further increase in CRP and SAA indicates acute systemic inflammation. During the later asymptomatic phase an increase in LCN-2 inflammatory biomarker and an increase in TNF-α leads to increased intestinal permeability affecting the tight junction integrity by decreasing claudin-1 with no signs of diarrhea and growth impairment.

In conclusion, our findings showed that EPEC infection causes growth impairment, diarrhea and increased inflammatory responses in weaned antibiotic pretreated mice. These effects were also dependent on an intact EPEC T3SS. In addition, metabolic perturbations and intestinal permeability were also observed in mice with EPEC infection, suggesting relevant biochemical pathways involved. Further, the findings presented here suggest that EPEC infections leads to an increase in intestinal and systemic inflammatory responses and transient overt diarrhea and growth impairment, as is often seen in children with EPEC infections. This EPEC infection model also presents two phases of diseases: an acute symptomatic and a later asymptomatic phase (**Figure 8**). This model can help further explore mechanisms involved in EPEC pathogenesis and perhaps facilitate the development of vaccines or therapeutic interventions.

## Author’s Note

This manuscript has been released as a pre-print at bioRxiv 2020.06.12.148593 (Ledwaba et al., 2020).

## Data Availability Statement

The raw data supporting the conclusions of this article will be made available by the authors, without undue reservation.

## Ethics Statement

The animal study was reviewed and approved by the Committee on the Ethics of Animal Experiments of the University of Virginia (Protocol Number: 3315). The mice used in the study have been handled with strict accordance with the recommendations in the Guide for the Care and Use of Laboratory Animals of the National Institutes of Health. All efforts were made to minimize suffering. This is also in accordance with the Institutional Animal Care and Use Committee policies of the University of Virginia. The University of Virginia is accredited by the Association for the Assessment and Accreditation of Laboratory Animal Care, International (AAALAC).

## Author Contributions

SL, DB, RG, JN, NP, and AT designed the project. SL, DC, and DB performed the experiments. SL, DC, DB, and RG analyzed the data and wrote the manuscript. PM assisted in analysis of results. NG and JS performed and assisted with metabolome analysis. All authors revised the manuscript. NP, AT, RG, and JN supervised the project. All authors contributed to the article and approved the submitted version.

## Conflict of Interest

The authors declare that the research was conducted in the absence of any commercial or financial relationships that could be construed as a potential conflict of interest.
