# Supplementary material for: Enteropathogenic Escherichia coli Infection Induces Diarrhea, Intestinal Damage, Metabolic Alterations, and Increased Intestinal Permeability in a Murine Model
Source: Front Cell Infect Microbiol. 2020 Dec 17;10:595266. doi: 10.3389/fcimb.2020.595266 (PMC7773950; doi:10.3389/fcimb.2020.595266)
Supplement: Supplementary file 1 [file DataSheet_1.docx]

**Supplementary Table 1:** Genes analyzed by Taqman qPCR.

| **Assay ID** | **Gene Symbol** |  | **Assay ID** | | **Gene Symbol** | |  |
| --- | --- | --- | --- | --- | --- | --- | --- |
| Mm99999915_g1 | Gapdh |  | Mm00434165_m1 | | Il12a | |  |
| Mm00446968_m1 | Hprt1 |  | Mm01288992_m1 | | Il12b | |  |
| Mm00446953_m1 | Gusb |  | Mm00434204_m1 | | Il13 | |  |
| Mm00431727_g1 | Agtr2 |  | Mm00434210_m1 | | Il15 | |  |
| Mm00432050_m1 | Bax |  | Mm00439619_m1 | | Il17a | |  |
| Mm00477631_m1 | Bcl2 |  | Mm00434225_m1 | | Il18 | |  |
| Mm00437783_m1 | Bcl2l1 |  | Mm00439620_m1 | | Il1a | |  |
| Mm00437858_m1 | C3 |  | Mm00434228_m1 | | Il1b | |  |
| Mm00839967_g1 | Ccl19 |  | Mm00434256_m1 | | Il2 | |  |
| Mm00441242_m1 | Ccl2 |  | Mm00434261_m1 | | Il2ra | |  |
| Mm00441258_m1 | Ccl3 |  | Mm00439631_m1 | | Il3 | |  |
| Mm01302428_m1 | Ccl5 |  | Mm00445259_m1 | | Il4 | |  |
| Mm99999051_gH | Ccr2 |  | Mm00439646_m1 | | Il5 | |  |
| Mm00438271_m1 | Ccr4 |  | Mm00446190_m1 | | Il6 | |  |
| Mm00432608_m1 | Ccr7 |  | Mm00434291_m1 | | Il7 | |  |
| Mm00515420_m1 | Cd19 |  | Mm00434305_m1 | | Il9 | |  |
| Mm00483137_m1 | Cd28 |  | Mm01328172_g1 | | Lrp2 | |  |
| Mm00519283_m1 | Cd34 |  | Mm00440227_m1 | | Lta | |  |
| Mm00483146_m1 | Cd38 |  | Mm00476361_m1 | | Nfkb1 | |  |
| Mm00599683_m1 | Cd3e |  | Mm00479807_m1 | | Nfkb2 | |  |
| Mm00442754_m1 | Cd4 |  | Mm00440485_m1 | | Nos2 | |  |
| Mm00441895_m1 | Cd40 |  | Mm00812512_m1 | | Prf1 | |  |
| Mm00441911_m1 | Cd40lg |  | Mm00478374_m1 | | Ptgs2 | |  |
| Mm00839636_g1 | Cd68 |  | Mm00448463_m1 | | Ptprc | |  |
| Mm00711660_m1 | Cd80 |  | Mm00441278_m1 | | Sele | |  |
| Mm00444543_m1 | Cd86 |  | Mm00441295_m1 | | Selp | |  |
| Mm01182107_g1 | Cd8a |  | Mm00448744_m1 | | Ski | |  |
| Mm00432688_m1 | Csf1 |  | Mm00489637_m1 | | Smad3 | |  |
| Mm00438328_m1 | Csf2 |  | Mm00484741_m1 | | Smad7 | |  |
| Mm00438334_m1 | Csf3 |  | Mm00782550_s1 | | Socs1 | |  |
| Mm00486849_m1 | Ctla4 |  | Mm00850544_g1 | | Socs2 | |  |
| Mm00445235_m1 | Cxcl10 |  | Mm00439518_m1 | | Stat1 | |  |
| Mm00444662_m1 | Cxcl11 |  | Mm00456961_m1 | | Stat3 | |  |
| Mm00438259_m1 | Cxcr3 |  | Mm00448890_m1 | | Stat4 | |  |
| Mm00487224_m1 | Cyp1a2 |  | Mm01160477_m1 | | Stat6 | |  |
| Mm00484152_m1 | Cyp7a1 |  | Mm00450960_m1 | | Tbx21 | |  |
| Mm00438656_m1 | Edn1 |  | Mm00441724_m1 | | Tgfb1 | |  |
| Mm00433237_m1 | Fas |  | Mm00443258_m1 | | Tnf | |  |
| Mm00438864_m1 | Fasl |  | Mm00437136_m1 | | Tnfrsf18 | |  |
| Mm01256734_m1 | Fn1 |  | Mm00449197_m1 | | Vcam1 | |  |
| Mm00442834_m1 | Gzmb |  | Mm00437304_m1 | | Vegfa | |  |
| Mm00772352_m1 | H2-Ea |  | Mm00802048_m1 | | Ace | |  |
| Mm00439221_m1 | H2-Eb1 |  | Mm00516023_m1 | | Icam1 | |  |
| Mm00516004_m1 | Hmox1 |  | Mm00434761_m1 | | Lif | |  |
| Mm00497600_m1 | Icos |  | Mm00444223_m1 | | Ly96 | |  |
| Mm00801778_m1 | Ifng |  | Mm01249194_m1 | | Nfatc3 | |  |
| Mm00833995_m1 | Ikbkb |  | Mm00452375_m1 | | Nfatc4 | |  |
| Mm00439616_m1 | Il10 |  |  |  | |  | |

**Supplementary Figure 1**

**

**

1. Levels of MPO on stools of control and EPEC mice were measured by ELISA on day 3 and day 7 post-infection (p.i.). Bars represent mean±SEM (n=8).

**Supplementary Figure 2**


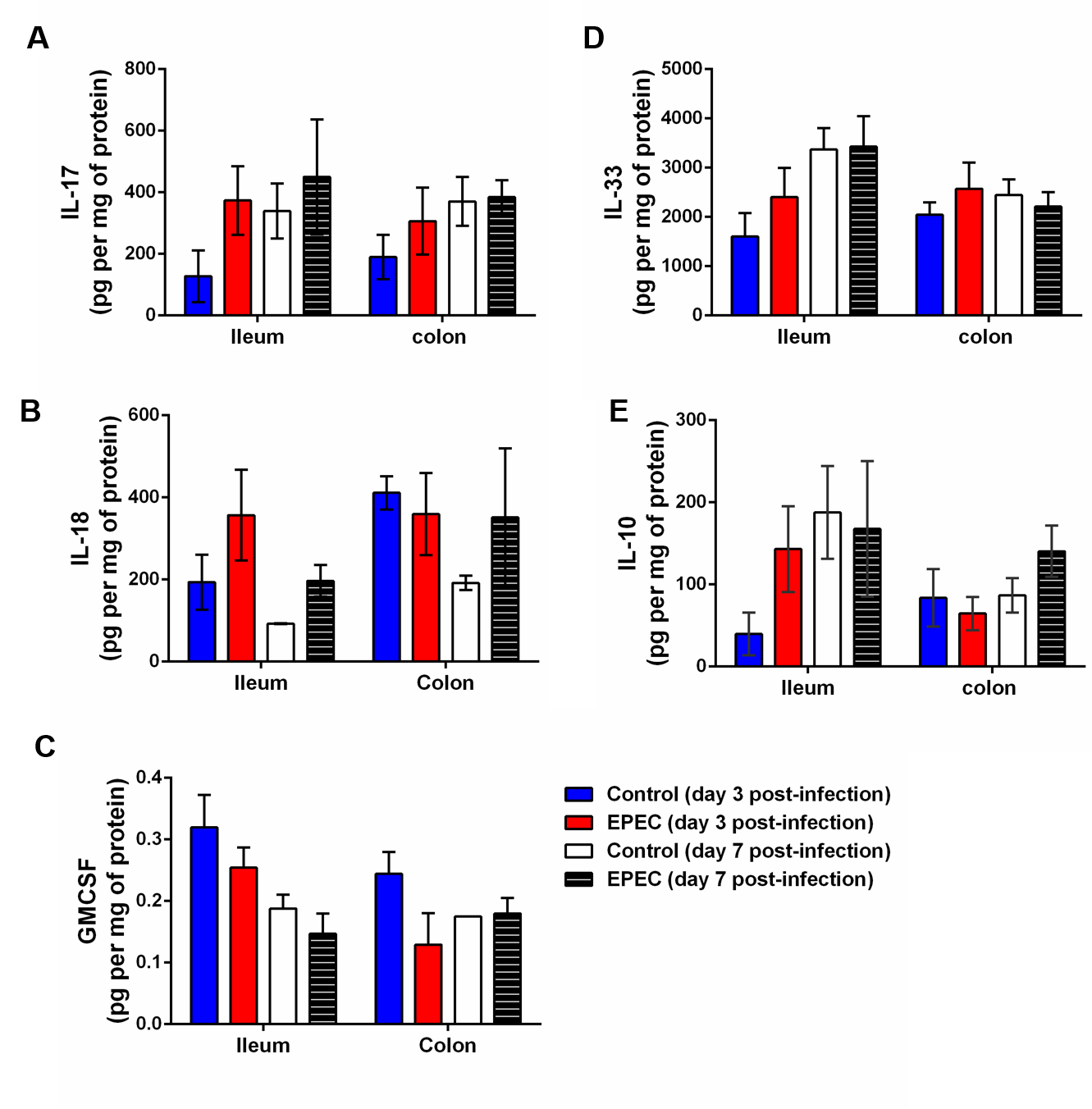


1. Levels of IL-17, **(B)** IL-18, **(C)**GMCSF, **(D)** IL-33 and **(E)** IL-10 in ileal and colonic tissues of control and EPEC infected mice were measured by ELISA at day 3 and 7 p.i. Bars represent mean±SEM (n=8).

**Supplementary Figure 3**


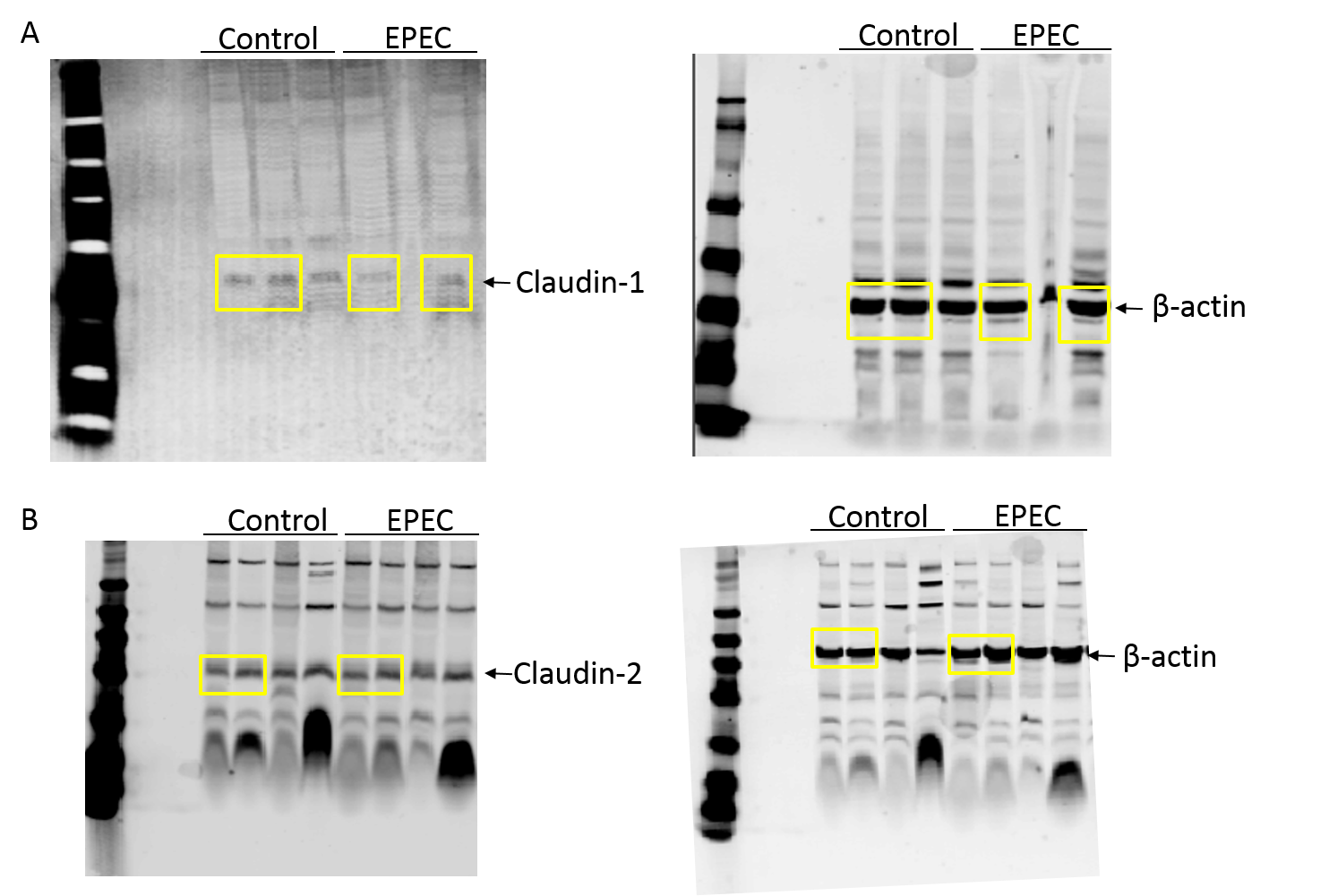


(A) Uncropped image of the blot used in figure 6C. (B) Uncropped image of the blot used in figure 6E. The dotted rectangle represents the cropped image used in figure 6C and 6E.

**Supplementary Figure 4**

**
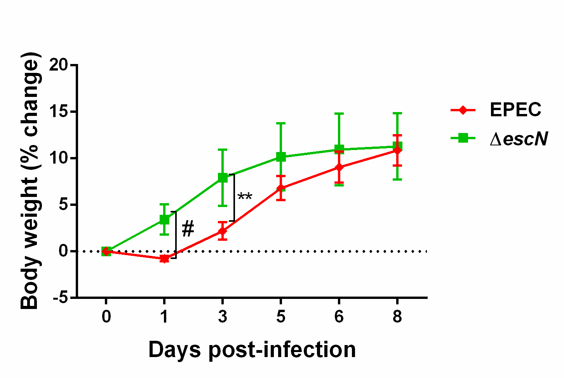
**

Change in body weight of weaned C57BL/6 mice infected with 10^10^ CFU per mouse with WT EPEC (EPEC) or Δ*escN* EPEC (Δ*escN*, T3SS deficient) (n=12/group). Line graphs represents mean±SEM. **p<0.001 and #p<0.05 using multiple Student’s *t-test*.
